# Supplementary material for: Splice-Junction-Based Mapping of Alternative Isoforms in the Human Proteome
Source: Cell Rep. Author manuscript; Available in PMC 2020 Jan 15. (PMC6961840; doi:10.1016/j.celrep.2019.11.026)
Supplement: 2 [file NIHMS1546469-supplement-2.zip › DF1/PXD010154/Thyroid/Thyroid_7_TG_RCPTPCQLQSEQAFLR.pdf]

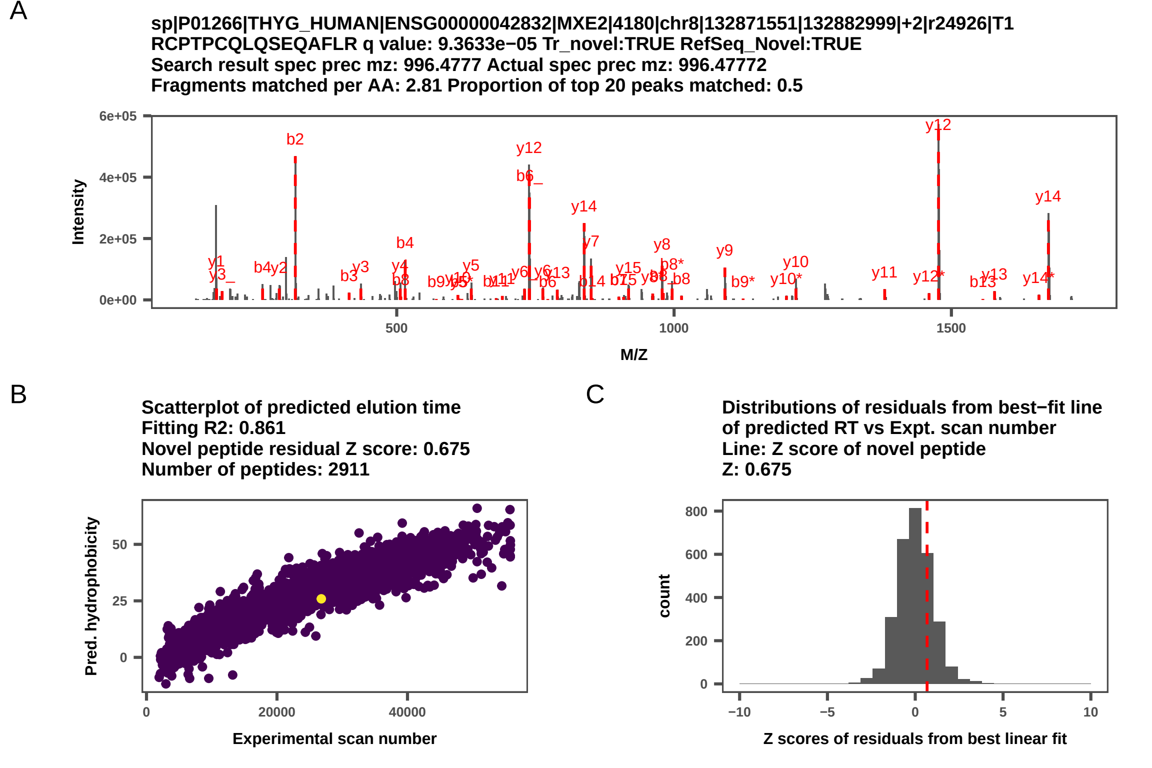
A
sp|P01266|THYG_HUMAN|ENSG00000042832|MXE2|4180|chr8|132871551|132882999|+2|r24926|T1
RCPTPCQLQSEQAFLR q value: 9.3633e−05 Tr_novel:TRUE RefSeq_Novel:TRUE
Search result spec prec mz: 996.4777 Actual spec prec mz: 996.47772
Fragments matched per AA: 2.81 Proportion of top 20 peaks matched: 0.5
Intensity
6e+05
4e+05
2e+05
0e+00
M/Z
500
1000
1500
b2
y12
b6_
y14
y7
b4
y1
y3_
b4y2
b3
y3
y4
b8
y5
y8
b8*
y9
y10
y10*
b9*
y11
y12*
y13
b13
y14*
y15
B
Scatterplot of predicted elution time
Fitting R2: 0.861
Novel peptide residual Z score: 0.675
Number of peptides: 2911
Pred. hydrophobicity
50
25
0
Experimental scan number
0
20000
40000
C
Distributions of residuals from best−fit line
of predicted RT vs Expt. scan number
Line: Z score of novel peptide
Z: 0.675
count
800
600
400
200
0
Z scores of residuals from best linear fit
−10
−5
0
5
10
